# Temporal stability of an endemic Mexican treefrog

**DOI:** 10.7717/peerj.1274

**Published:** 2015-09-24

**Authors:** Griselda Cruz-Ruiz, Crystian S. Venegas-Barrera, Hermilo Sanchez-Sanchez, Javier Manjarrez

**Affiliations:** 1 Facultad de Ciencias, Universidad Autónoma del Estado de México, Toluca, Estado de México, México; 2 División de Estudios de Posgrado e Investigación, Instituto Tecnológico de Ciudad Victoria, Ciudad Victoria, Tamaulipas, México

**Keywords:** Population abundance, Sex ratio, Treefrog, Size structure, *Hyla*

## Abstract

The demographic characteristics of an amphibian population fluctuate independently over time, mainly in response to the temporal variation of environmental factors, especially precipitation and temperature. These temporal fluctuations may contribute to the size of an amphibian population and could be used to determine the current conservation status of a species. During a five year (2004–2008) period, we studied the relative abundance, sex ratio, and age-sex structure of a population of metamorphosed individuals of the endemic treefrog *Hyla eximia* in Central Mexico. We also studied the species’ relationship with climatic variables such as temperature and precipitation. We found an interannual constant abundance during the study period. However, interannual differences were observed in the population structure by age-sex category (males, females, or juveniles), with decreased abundance of males and juveniles during the rainy months (August–November). The annual abundance of *H. eximia* was positively correlated with rainfall, but negatively with monthly temperature. We found the sex ratio was male-biased (2:1), except for year 2008. Also, differences in snout-vent length (SVL) were found between years, suggesting changes in recruitment of new individuals. We conclude that variations in abundance, and frequencies by age-sex category, of *H. eximia* are related to seasonal variations in temperature and precipitation characteristics of temperate zones. However, this temporal stability may suggest that anurans have an unusual capacity to persist even in the face of human-induced habitat change.

## Introduction

Recent reports suggest that amphibians can be particularly sensitive to human impacts because of certain features of their biology and ecology (Pechmann & Wilbur, 1994; Blaustein et al., 2011; Collins & Storfer, 2003). Although these reports describe the amphibian decline as a recent worldwide phenomenon, local population declines are less evident because declines are common features of many apparently undisturbed populations (Pechmann & Wilbur, 1994). In addition, long-term data on the population dynamics of amphibians are unavailable for most cases of reported declines. These studies may serve as a baseline for comparisons or may reveal that many of the reported declines are indeed unprecedented in ecological time because much of what is known about amphibian population dynamics comes from relatively brief studies or anecdotal accounts (Blaustein, Wake & Sousa, 1994).

Amphibian population sizes naturally fluctuate within wide margins (Joseph, Pechmann & Wilbur, 1994; Houlahan et al., 2000) and it can be difficult to determine the cause of a population decrease because disturbances such as habitat loss, disease, and climate variations can play a large role. It is necessary to have long-term data to reliably determine the temporal stability of an amphibian population (Kiesecker, Blaustein & Belden, 2001).

Population size is a key parameter to assessing the conservation status of a species and changes in population size or structure may give us clues about its current status. It is also recognized that parameters such as sex and age of individuals contribute differentially to the effective size of amphibian populations (Lee, Saether & Engen, 2011).

In amphibians, population variations are determined primarily by patterns of precipitation and temperature (Blaustein et al., 2010; Rohr & Palmer, 2013).Their physiological characteristics make them highly dependent on temperature and humidity for maintenance of fitness functions (Duellman & Trueb, 1994; Pough et al., 2003). This dependence on abiotic environmental conditions is reflected in both the start and duration of reproductive activity and survival (Blaustein et al., 2010).

Moreover, the sex ratio (number of males: number of females) determines patterns of competition, mate choice, and reproductive potential of a population (Krebs, 2009). A bias in the sex ratio leads to variations in reproductive events which may cause the loss of genetic variability, the fixation of mutations, and reduced population size (Cotton & Wedekind, 2009), which may in turn increase the risk of extinction of small, isolated populations (Stelkens & Wedekind, 2010).

The age and size of individuals can also affect the dynamics of populations. While young individuals usually have lower survival rates, the recruitment of youth is key to maintaining a population and is an important measure of its conservation status. High recruitment rates may act to offset lower survival rates caused by adverse environmental conditions (Muths, Scherer & Pilliod, 2011).

Interpreting the effect of environmental changes on population dynamics is necessary to identify temporal and spatial variations of population parameters (Coulson et al., 2001). Obtaining this information is important in planning and evaluating population studies (Hiert & Moura, 2010; Gillespie, 2011), and for the maintenance and conservation of amphibian populations. For example, knowing the temporal activity pattern of a species is useful for determining optimal times to conduct studies, population inventories, and maintenance and conservation activities.

Hylidae species are experiencing rapid population declines (Stuart et al., 2004). In Mexico, 61 anuran species are listed as endangered, and of these, 43 (70.5%) belong to the family Hylidae (NOM-059-ECOL-2010). The endemic mountain treefrog, *Hyla eximia,* is partially sympatric with *H. plicata* (Smith et al., 2007) in parts of the Mexican Plateau. Both species occupy the same mating ponds in a 500-m altitudinal band (2,400–2,900 masl) where their distribution ranges overlap, but only *H. plicata* is considered threatened (NOM-059-ECOL-2010). *H. eximia* is a species classified as “least concern” by the IUCN Red List of Threatened Species (2015) because of its wide distribution and presumed large population. However, the lack of studies, and little knowledge of their ecology and distribution in the Central Mexican Plateau, where some of the highest human-populated zones of México exist, signals a potentially dangerous scenario for this species (Hammerson & Canseco-Márquez, 2010).

The aim of this study is to describe the abundance, age-sex structure, and sex ratio of a population of mountain treefrogs, *H. eximia*, on the Central Mexican Plateau. We associate these population parameters with environmental factors of temperature and precipitation to determine if there are significant annual variations in the population structure over a period of five consecutive years. This information can help scientists better understand fluctuations in populations of *H. eximia* and can be used to prioritize and implement conservation efforts.

## Methods

This study received the approval of the ethics committee of the Universidad Autónoma del Estado de México (number 3589/2013SF). All subjects were treated humanely on the basis of guidelines outlined by the Society for the Study of Amphibians & Reptiles.

The study population is located within an oak forest of El Pedregal de Guadalupe Hidalgo (19°14′N, 99°27′W; 2,650 m altitude) in the city limits of Ocoyoacac, State of México, on the Central Mexican Plateau. The region has a sub-humid temperate climate with an average annual temperature of 18 °C. Annual rainfall is between 1,400 and 1,800 mm. The area remains dry from December through May, but during the rainy season intense rainfall occurs from July through August, forming a temporary pond approximately 2.6 km^2^ which usually remains until November.

We visited this temporary pond once every two weeks from June through November for five consecutive years (2004–2008) to record the number of individuals that we could see as we walked around the margin of the pond. Each visit was conducted by one person between the hours of 1000 and 1400 (CDT). We only sampled during the day because this study was not focused on the reproductive activity of *H. eximia*. At the end of each visit, some frogs were captured from around the margin of the pond. We recorded their snout-vent length (SVL in mm), length of tibia (Lt in mm), and body weight (g). Where possible, the sex of each individual was determined by observation of external sexual characteristics as in Duellman (2001). Males present a dark sub-gular sac as an external secondary sexual characteristic, while females lack this pigmentation and have a light-colored belly with homogeneous pigmentation. The reliability of this visual sex determination was verified by dissection (presence of ovaries or testes) of 47 frogs collected during the summer in a previous study of the same population. These 47 frogs were also measured for SVL, Lt, and reproductive or non-reproductive status.

Temperature and rainfall data were obtained from daily records of the La Marquesa weather station (19°18′N, 99°02′W, 3,060 m altitude). For statistical analysis, we used monthly accumulated precipitation and monthly maximum temperature because they were the only records available from the weather station.

## Statistical Analysis

**Figure 1:**
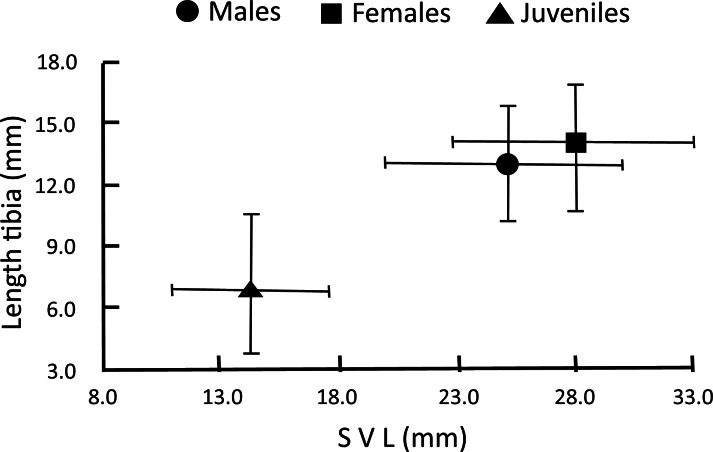
Age-sex categories of *H. eximia* defined by the generalized analysis of k-means on sex-body size. Juveniles (SVL < 20 mm), adult males and adult females (SVL ≥ 20 mm).

Because SVL is not a good indicator of the age of metamorphosed individuals (Duellman & Trueb, 1994), we grouped the captured frogs into three age-sex categories: juveniles (SVL < 20 mm), adult males (SVL ≥ 20 mm and dark sub-gular), and adult females (SVL ≥ 20 mm, light sub-gular; Fig. 1), as defined by the generalized analysis of k-means. This analysis grouped the frogs in k-groups based on three continuous variables (SVL, Lt, and body weight) and color of sub-gular sac (dark or light) as a categorical variable. The number of groups was previously defined by cluster analysis using agglomerative Euclidean distance and Ward amalgamation algorithm. For data obtained from the dissection of individuals, a Kruskal–Wallis test was performed to compare the SVL.

We performed ANOVA analysis to identify interannual variations (predictor variable) in body size (outcome variable) for each sexual category (separate models). The reduction in mean could indicate recruitment of recently transformed individuals in each sexual category (Donnelly, 1999). The annual sex ratio and possible interannual differences were explored with a chi-square test assuming a 1:1 sex ratio.

We measured the monthly abundance of frogs by adding up the number of individuals viewed per month in the pond. Frogs were not marked and so many of the same individuals were counted every time, but this possible bias should be accounted for in the data analysis (Wells, 2007). These data were adjusted by a quadratic transformation to achieve normality according to that described by Hiert & Moura (2010) and Zar (1984).

We used a correspondence multi-factor analysis to explore the association of the frequency of observed frogs according to three categorical variables: body size (two categories, juvenile and adult), sex (two categories, male and female) and month (six categories, from June to November). The analysis explored the structure of the combination of all these categories in a contingency table through the generation of new variables (dimensions) that summarize the differences among the three variables. The first dimension accounts for the largest variance and successive dimensions reflect a lower variance (Gotelli & Ellison, 2004). We interpreted our results only with dimensions (identified with Chi-square test) that contributed significantly to explaining the variance observed (Rencher, 2002). As each dimension was orthogonal, we generated a scatter plot with two dimensions to represent the separation of the categories according to frequency of frogs. Scatter plots show the association among categories, where close coordinates (categories of different variables) indicate a high relative frequency of frogs than other combinations. Categories with larger variations will be far from centroid (0, 0 coordinate) than nearby coordinates. The null hypothesis to be tested was the frequency of frogs, depending sex and body size, did not vary across months. This hypothesis was contrasted with an alpha value of 0.05

To explore interannual variation in rain or temperature, we used the Kruskal–Wallis test with monthly maximum temperature and accumulated monthly rainfall. Finally, these two variables were correlated with the annual relative abundance of frog with nonparametric Spearman correlation. Body sizes (SVL) are reported as mean ±1SD.

## Results

### Population structure

Adults showed sexual dimorphism, with females having a larger SVL (28.14 ± 3.11 mm) than males (25.3 ± 2.02 mm; Kruskal–Wallis *H*_2_ = 661.86, *p* = 0.001; Fig. 1). Of dissected *H. eximia*, adult females were gravid with abdominal free oocytes, testicles of adult males showed developed fatty bodies, and juveniles had immature sexual organs. These results suggest that sexual maturity and morphological differentiation is achieved when individuals reach a SVL of greater than 20 mm. This confirms our classification of individuals as described in the Methods section.

**Figure 2:**
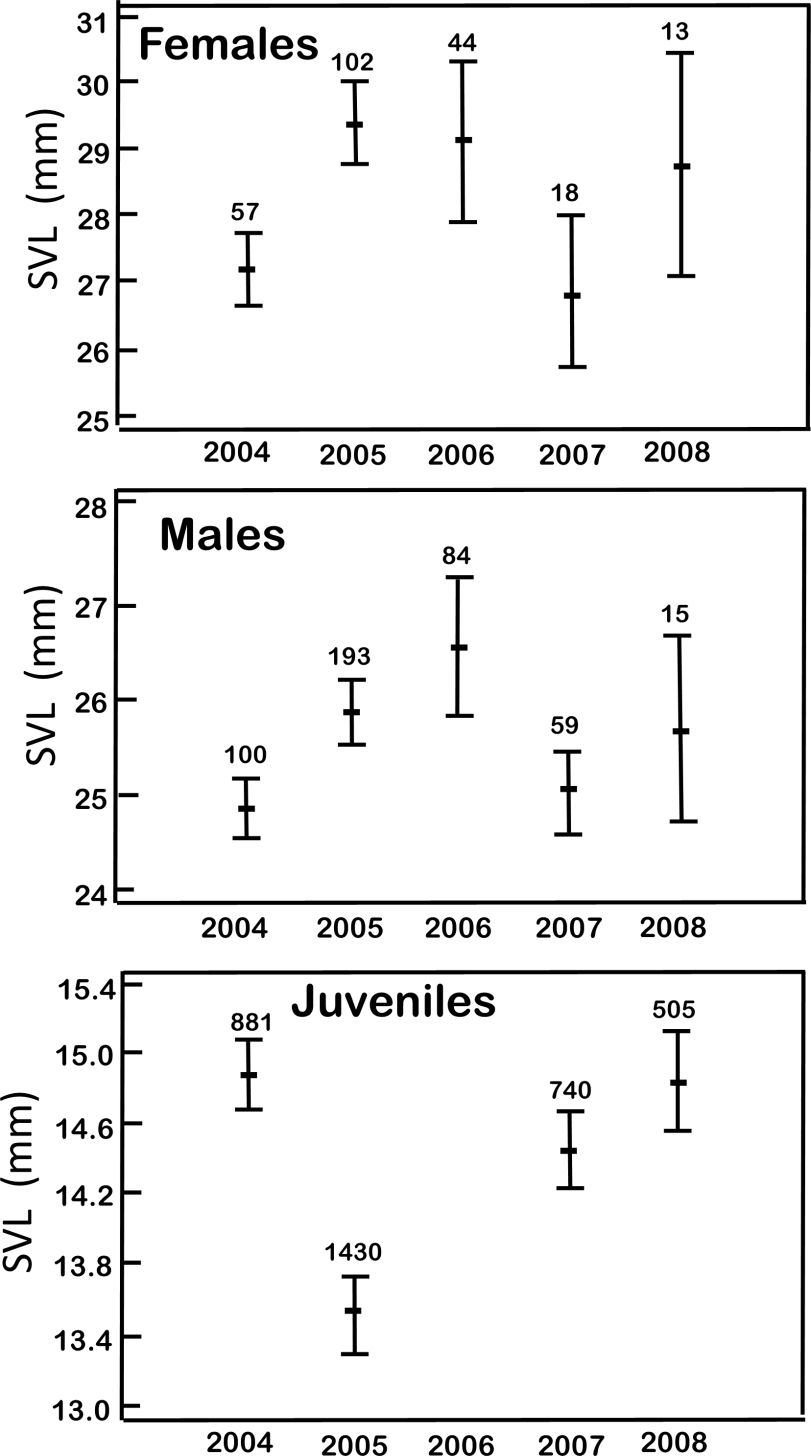
Mean body size (SVL ± 1SD) in the age-sex categories of *H. eximia* during the five-year study at Ocoyoacac, State of Mexico.

The body size in the age-sex groups showed differences among the five years of records (ANOVA: females *F*_4,229_ = 4.39, *p* = 0.002; males *F*_4,446_ = 3.99, *p* = 0.003; juveniles *F*_3,3553_ = 19.35, *p* = 0.0001; Fig. 2). In 2007, the females had a lower SVL (26.87 ± 3.39 mm) than other years; 46% of females ranged from 20.00 to 25.41 mm SVL. The predominant body size in the other years ranged from 25.41 to 36.0 mm SVL. The males had the lowest SVL in 2004 (24.87 ± 1.87 mm) when 94% of males were between 24 and 28 mm. For juveniles, the lowest SVL occurred in 2005 (13.51 ± 2.04 mm) with 93% of juveniles between 10.3 and 16.65 mm.

**Figure 3:**
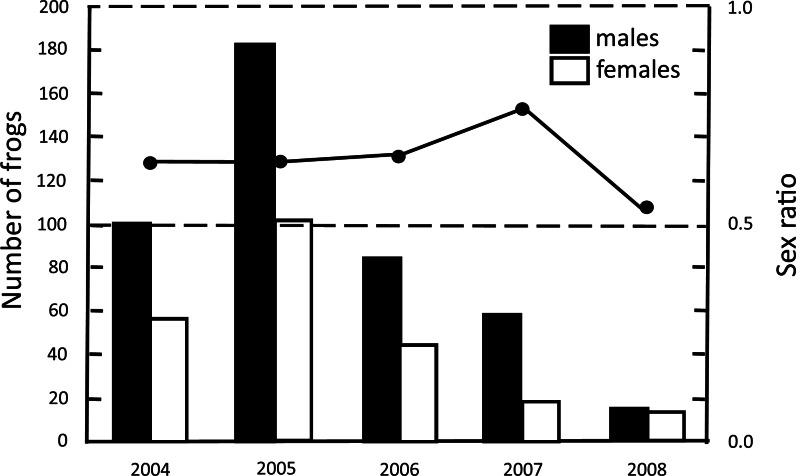
Abundance of adults and sex ratio (proportion of males) of *H. eximia* during the five-year study at Ocoyoacac, State of Mexico.

The sex ratio (2:1) was skewed towards males during four years of records (Fig. 3), but in 2008 the sex ratio was close to 1:1 (${X}_{1}^{2}=0.033,p> 0.05$; Fig. 3).

### Temporal abundance

**Figure 4:**
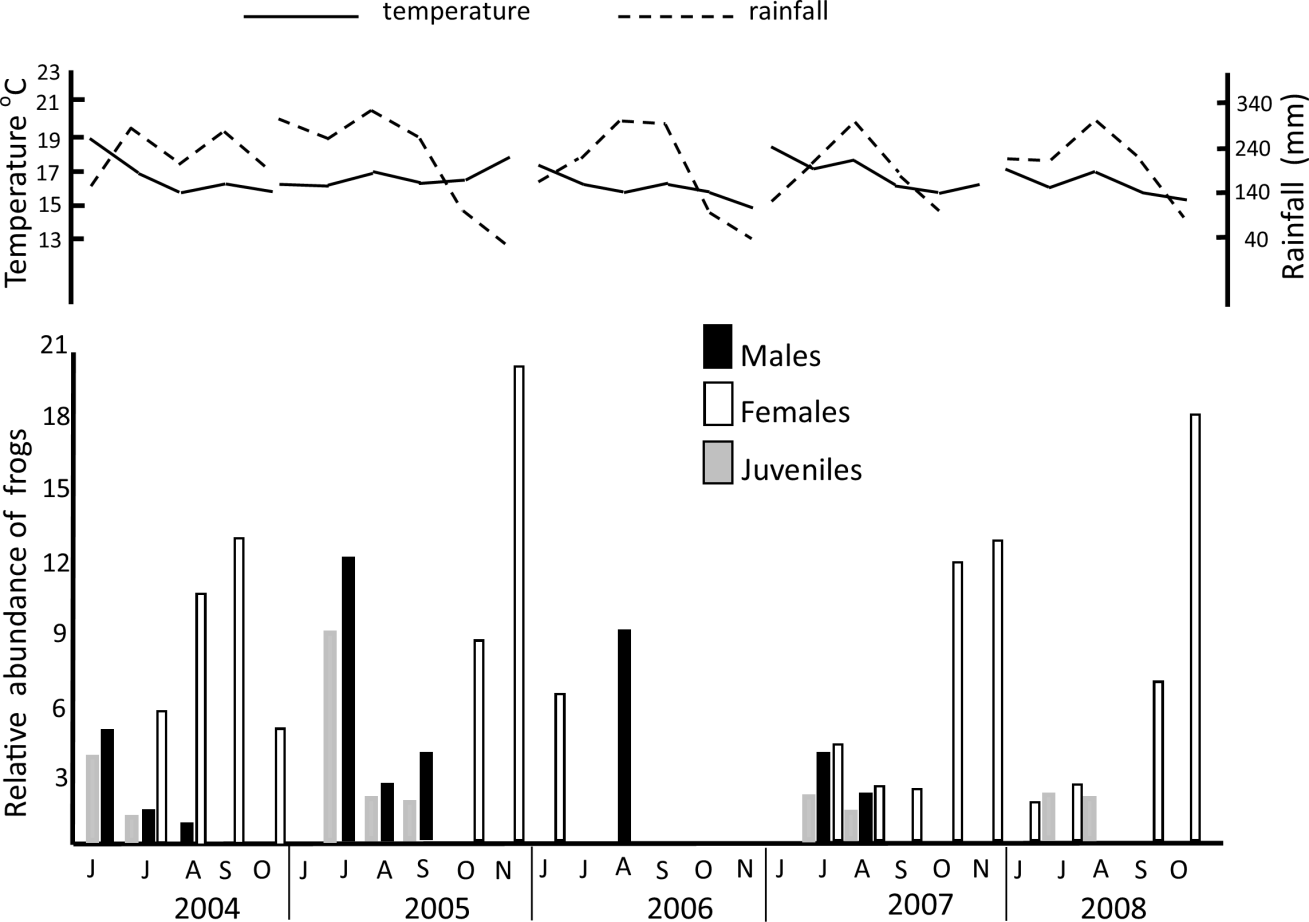
Annual relative abundance of *H. eximia*, total precipitation, and maximum temperature at Ocoyoacac, State of Mexico, during activity season by each year of study.

During the five years of study, a total of 4,241 frogs were counted in the pond with similar abundances between years (Kruskal–Wallis *H*_4_ = 2.64, *p* = 0.451). The frogs were visually present for 5 to 6 months each year starting in June and ending in early November (Fig. 4). In general, the highest abundance was recorded from August to November, but showed monthly and yearly variation (Kruskal–Wallis *H*_5_ = 11.9, *p* = 0.036, Fig. 4).

Correspondence analysis showed seasonal variations in frequency of age-sex groups observed in the pond $({X}_{42}^{2}=3575.90,p=0.0001)$. Adults of both sexes occurred more frequently from July to September. In the later months (August to November), males and juveniles decreased, while females became more abundant. Although this seasonal pattern shows some variation between the years of study, the Kruskal–Wallis test showed no significant differences in abundance by age-sex groups between years (juveniles *H*_3_ = 1.45, *p* = 0.693; females *H*_4_ = 1.05, *p* = 0.83; males *H*_4_ = 1.83, *p* = 0.76).

Considering the months that we observed frogs, the annual relative abundance of *H. eximia* was positively correlated with rainfall (Spearman *r*_28_ = 0.50, *p* = 0.0001), but negatively correlated with monthly maximum temperature (Spearman *r*_28_ = − 0.07, *p* = 0.0001; Fig. 4). Variations in the rainfall and monthly maximum temperature are seasonal in nature. The most notable differences were observed in periods marked by low rainfall from December to April and periods of heavy rain August and September (309.72 and 267 mm, respectively). The monthly maximum temperature ranged from 13.72 °C to 20.76 °C. The highest temperatures occurred in March and April (18.77 °C and 19.95 °C, respectively). Comparisons between years for both variables show no difference (temperature: *H*_59_ = 0.69, *p* = 0.951; rain: *H*_59_ = 1.24, *p* = 0.87).

## Discussion

Amphibian populations naturally fluctuate within wide margins (Joseph, Pechmann & Wilbur, 1994; Houlahan et al., 2000). For example, during a 15-year study, the abundance of the Pacific tree frog (*Pseudacris regilla*) varied from 1 to 126 individuals in northwestern Nevada (Weitzel & Panik, 1993). To observe the actual range of fluctuations in abundance, long-term studies are recommended (Lips et al., 2001).

In our five-year study we did not observe significant interannual variations in the abundance of frogs. Stability in the abundance of a population must also take into consideration survival and longevity of adult individuals (Wells, 2007) that act as a buffer to the negative effects of larval mortality and emigration rate during dry periods (Price, Browne & Dorcas, 2012). The male:female sex ratio of 2:1 is consistent with that of most species of frogs (Duellman & Trueb, 1994). There are numerous possible causes for this bias, some of which include, late sexual maturation of females, the postponement of reproduction in females, and a possible differential survival between the sexes (Wells, 2007). Another possible source of bias, is sex-biased spatial distribution (Pröhl & Berke, 2001; Johnson, Knouft & Semlitsch, 2007).

For all age-sex categories, we observed significant variations in body size. In 2005 smaller juveniles were recorded, but this was also the year with the highest number of individuals (1,430). This could indicate that in 2005 there was greater success in the survival of the larvae. Among adults, we observed sexual dimorphism, with females larger than males, similar to that observed in 90% of all species of Anura (Shine, 1979). These differences in size could be related to differences in age between the sexes, and the fecundity of the females (Monnet & Cherry, 2002).

The abundance of amphibian species that breed in temporary bodies of water, such as *H. eximia*, is dependent on the time of flooding and the permanence of the water body. In this study, the *H. eximia* population showed the characteristic pattern of temporary abundance during the wet season, which is characteristic of other Anuran populations in temperate zones with marked seasonality of rainfall. However, our results of abundance are likely biased by the sampling during day, when reproductive activities were possibly excluded.

This seasonality marks the beginning of reproduction (late May through early June) as well as differences in the abundance by age-sex categories. During June and July, when the pond began to form, the frequency of adults in the pond was low. During August through November, the number of adult females was greater, while the number of males and juveniles decreased (Fig. 4). August usually had the highest rainfall and tapers off fast from September to November, the start of the dry season. No unusual environmental conditions (prolonged drought or frost) were observed during the five years of study. The absence of juveniles later in the season could indicate that they grow to adult size by September or October. This possibility is feasible because growth rates in juvenile treefrog, *Hyla cinérea* range from 0.17 to 0.42 mm/day, equivalent to 5.1–12.6 mm/month, growing under laboratory conditions (after metamorphosis and adjusted for temperature and size at metamorphosis; (Blouin, 1992)), and similar results were reported for *H. regilla* in natural conditions with an average growth somewhat less than 8 mm/month (Jameson, 1956).

In this study, no differences were found in the yearly abundance of *H. eximia*, but the analysis of SVL gives an indication of possible changes in the age-size structure. It would be advantageous to complement the study with other techniques to establish the age and size of individuals entering the adult population.

## Conclusions

Changes in abundance and distribution of the three *H. eximia* age-sex categories, are related to seasonal variations in temperature and precipitation during the year. The ability of *H. eximia* to persist is constrained by human-induced habitat alterations of the temperate forest habitat in the Central Mexican Plateau. Small frog species, such as *H. eximia*, may be strongly disadvantaged by habitat fragmentation. The Central Mexican Plateau contains a major metropolitan area and 44.7% of this area is used for agriculture (CONAPO, 2010). This presents the possibility of a reduction in the suitability of existing habitat patches (i.e., fragments) which may affect both local and landscape-level pond-breeding amphibian population dynamics. Only temperate forest habitat seems to be suitable for *H. eximia*, therefore, conservation efforts should be directed to areas where suitable ecological variables are present and especially where human encroachment affects tree microhabitat.

Most of what we know about this endemic treefrog comes from occasional captures of active animals. New studies are needed to better understand how the stability of a population of frogs relates to areas that are degraded by human land-use. For example, mark–recapture studies can elucidate terrestrial habitat requirements for local populations, genetic approaches may be used to infer gene flow, and to estimate dispersal and immigration. These types of studies will provide crucial information about the relative impact of land use on effective population size and reproductive success of local populations (Johnson, Knouft & Semlitsch, 2007).

## Supplemental Information

10.7717/peerj.1274/supp-1 Table S1 Data collected of *H. eximia* at Ocoyoacac, State of Mexico, during five years of study
